# A role for autonomic cardiac control in the effects of oxytocin on social behavior and psychiatric illness

**DOI:** 10.3389/fnins.2013.00048

**Published:** 2013-04-02

**Authors:** Daniel S. Quintana, Andrew H. Kemp, Gail A. Alvares, Adam J. Guastella

**Affiliations:** ^1^SCAN Research and Teaching Unit, School of Psychology, The University of SydneySydney, NSW, Australia; ^2^Brain and Mind Research Institute, The University of SydneySydney, NSW, Australia

**Keywords:** oxytocin, autonomic nervous system, heart rate variability, social cognition, social behavior

## Abstract

Cumulative evidence over the last decade indicates that intranasally administered oxytocin (OT) has a major impact on social behavior and cognition. In parallel, researchers have also highlighted the effects of OT on cardiovascular (CV) and autonomic nervous system (ANS) regulation. Taken at face value, these two streams of research appear largely unrelated. However, another line of evidence highlights a key role for autonomic cardiac control in social behavior and cognition. In this review, we suggest that autonomic cardiac control may moderate the relationship between OT and social behavior. We also highlight the importance of autonomic cardiac control in psychiatric disorders of social dysfunction and suggest that heart rate variability (HRV)—an index of autonomic cardiac control—may play a key role in patient response in treatment trials of OT.

## Introduction

Over the past decade there has been a growing body of research on humans demonstrating the social effects of oxytocin (OT) nasal spray on social cognition and behavior. OT is related to social behaviors such as trust (Kosfeld et al., 2005), increased gaze to the eye region of the face (Guastella et al., 2008), and emotion recognition (Domes et al., 2007a). In addition, OT has been found to modulate social distance between males and females (Scheele et al., 2012), increase memory for facial identity (Savaskan et al., 2008), increase attention to social cues (Leknes et al., 2012), motivate in-group cooperation (De Dreu, 2012), and increase generosity (Zak et al., 2007). Along with these interesting effects there is a growing interest in understanding how these effects occur in humans and what may represent critical markers of this response (Guastella and MacLeod, 2012).

Although OT is a relatively “new” hormone in the social neuroendocrine field, its role in cardiovascular (CV) activity and autonomic nervous system (ANS) function more generally has been recognized since the 1940s. Early work by Woodbury et al. demonstrated that OT reduces blood pressure in humans (Woodbury and Abreu, 1944; Woodbury et al., 1944). In these experiments, OT was administered intravenously (in males) or into the wall of the uterus (in non-pregnant and pregnant females). The authors reported that OT administration reduced arterial pressure and corresponding tachycardia, between 1 and 5 min after administration. More recently, animal research has demonstrated that OT is also involved in attenuating stress-induced heart rate (HR) increases (Grippo et al., 2009), the modulation of breathing (Mack et al., 2002), and bradycardia via increases in vagal outflow (Higa et al., 2002). Superficially, the role of OT on social cognition and its involvement in the regulation of CV systems appear unrelated. However, there is growing recognition for the critical role of CV systems in stress regulation, social approach, attachment, and empathy. Thus, the purpose of this review is to integrate research on OT from these seemingly disparate fields to (1) explain the physiological basis of social behavior and stress focusing on heart rate variability (HRV) in particular; (2) review the evidence for an important relationship between OT, its administration, and CV function; (3) highlight studies that link CV function to social processes, including social cognition, attachment, empathy, and social dysfunction in psychiatric disorders, and (4) propose that HRV may provide a marker of response to OT treatment to predict who might respond favorably to its administration to improved social capacity in humans.

## Autonomic cardiac control, survival, and social behavior

Autonomic cardiac control plays a crucial role in social behavior (Porges, 2001) and attachment (Porges, 2003). To explain such observations, Porges proposed the Polyvagal theory (2001; 2011) to argue that humans have evolved a dynamic regulatory system enabling adaptive responsiveness to safe, dangerous or life-threatening events and contexts. When an organism is under threat, the “vagal brake” is released facilitating a significant amount of energy expenditure that promotes survival. However, during times of safety, the ANS promotes approach-related behaviors. The nucleus of the solitary tract in the brain stem is an important relay station for both the regulation of the ANS and the control of facial muscles as it contains fibers from both the facial (Nageotte, 1906) and the vagus nerves (Pearson, 1947). Due to these tightly integrated networks the ANS is well-placed to influence emotional expressivity (via facial expressions and vocalizations) along with the perception of emotion (via the control of eyelid opening and eye gaze to see emotional stimuli and middle ear muscles to detect subtle emotional nuances in human voices).

A key facet of the “global” social ANS is a neural circuit, the social engagement system (Porges, 2001). This system is controlled by the cortex, which regulates responses from brainstem nuclei governing a number of responses crucial for social communication (e.g., facial expression). This system facilitates strong social bonds for caregiver–child attachment and relationships across the lifespan (Porges, 2003). The social engagement system is comprised of visceral efferents and the myelinated vagus. This system is unique from other parts of the social ANS as the cranial nerves that regulate this system (i.e., cranial nerves V, VII, IX, X, and XI) developed together embryonically (Porges, 1998). Efferent cranial nerve traffic regulates facial muscles (e.g., emotional expression), laryngeal and pharyngeal muscles (e.g., vocal prosody), and eyelid opening (e.g., looking at social stimuli). Importantly, the source nuclei of these nerves found in the brainstem directly communicates with the visceromotor part of the nucleus ambiguus. This portion of the nucleus ambiguus is the source nuclei of an inhibitory element of the ANS. This inhibitory system has been theorized to promote a calm, restorative state by facilitating a slower HR and lower blood pressure and reducing sympathetic activity via the myelinated vagus to the sinoatrial node, which is the heart's pacemaker (Porges, 2001). As well as regulating social behavior between adults, this inhibitory system also supports attachment behaviors between child and caregiver (Porges, 2003). A lack of secure emotional attachment from a caregiver may impair biobehavioral systems later in life that underpin social behavior (Fries et al., 2005).

The heart is dually innervated by both branches of the ANS; an increase in HR is associated with greater sympathetic influence whereas a decrease in HR is associated with greater parasympathetic influence. However, HR alone is a poor index of ANS activity. An increase in HR, for example, could be attributed to a combination of reduced parasympathetic activity and increased sympathetic activity. Early conceptualizations of the ANS by Langley (1921) and Cannon (1939) suggested that the sympathetic nervous system (SNS) and parasympathetic nervous system (PNS) work in opposition insofar that if one system exerts dominance, the other system correspondingly retreats. While there are instances, such as baroreflex activation (Kollai and Koizumi, 1979) that are consistent with this “classical” interpretation, more recent research since has revealed that both branches of the ANS can operate independently of one another (Berntson et al., 1991). In other words, the two branches of the ANS can perform in a reciprocal fashion (i.e., sympathetic activation corresponds with parasympathetic withdrawal and vice versa), the PNS and SNS can be co-activated or co-inhibited, or the PNS and SNS can operate uncoupled.

## Autonomic cardiac control, heart rate variability, and motivation for social engagement

HRV is a non-invasive and relatively inexpensive index of autonomic cardiac control (Berntson et al., 1997) that can be measured by the interbeat intervals derived from an electrocardiogram (ECG). Derived from power spectral analysis of interbeat intervals, high frequency HRV is a relatively pure measure of PNS activity (Berntson et al., 1997). We have recently proposed that resting state HRV may reflect one's capacity for social-approach and motivation for social engagement (Kemp et al., 2012a,b). Data from Kok and Fredrickson (2010) indicates that high HRV facilitates greater chances for social opportunities, which then leads to high HRV, leading to an “upward spiral” of reciprocal causality. In addition, increases in resting state HRV measured between the ages of 14 and 16 has been found to predict increases in behavioral warmth (e.g., joyful approach behaviors, empathic understanding) between the same age periods (Diamond and Cribbet, 2012). In adults, high HRV has also been associated with an increased number of self-reported positive social interactions in co-habiting couples (Diamond et al., 2011) and predicts relationship attachment quality (Diamond and Hicks, 2005). For example, supportive relationships have been related to higher HRV in comparison to ambivalent relationships (Holt-Lunstad et al., 2007), highlighting the importance of HRV in approach related behaviors.

By contrast HRV is reduced in a number of psychiatric disorders that are characterized by poor social functioning and social withdrawal. For example, we have previously reported that HRV is reduced in depression (Kemp et al., 2010) and alcohol dependence (Quintana et al., 2013), and these findings were not due to a prior history of CV disease. We have also demonstrated that patients with depression and comorbid generalized anxiety disorder display the greatest reductions in HRV and these findings were independent of depression severity (Kemp et al., 2012a,b). Others have also shown that HRV is reduced in autism spectrum disorders (ASD; Bal et al., 2010) and first episode psychosis (Jindal et al., 2009). While the incidence of CV disease is higher in psychiatric illness than in controls without such illness (Colton and Manderscheid, 2006; Goodwin et al., 2009), many studies on HRV have reported findings based on patients without any history of CV disease. These HRV impairments in psychiatric populations may be interpreted in regards to self-regulatory capacity (Segerstrom and Nes, 2007) and impulse control (Allen et al., 2000; Quintana et al., in press). HRV reductions may also reflect an early indicator of future morbidity and mortality from a host of conditions (Thayer and Brosschot, 2005; Thayer and Sternberg, 2006; Thayer and Lane, 2007; Thayer et al., 2010).

## Autonomic cardiac control regulates the perception and projection of emotional cues

Psychological processes such as emotion and cognition are underpinned by a common reciprocal inhibitory cortico-subcortical neural circuit—the central autonomic network—and activity in this circuit can be indexed by HRV (Thayer, 2009). Social behavior may therefore be limited by an individual's physiological state leading to a number of predictions.

First, a calmer physiological state characterized by increased parasympathetic activity will facilitate the *perception* of emotion. A recent meta-analysis of neuroimaging studies revealed an association between autonomic cardiac control and the dorsomedial prefrontal cortex in particular—an important brain region for emotion recognition and social cognition (Thayer et al., 2012). There is a high degree of connectivity between brain structures that regulate autonomic cardiac control and the perception of emotion (Smith and DeVito, 1984; Thayer et al., 2009). The brain stem operates as a relay station between the prefrontal cortex, involved in the conscious perception of emotion, and structures that regulate autonomic cardiac control, such as the nucleus of the solitary tract (Smith and DeVito, 1984; Thayer et al., 2009). Given the close relationship between these central structures it is hypothesized that tasks leading to vagal withdrawal will also impact on the perception of emotion.

In regards to this first prediction, we recently investigated the relationship between resting state HRV and emotion recognition (Quintana et al., 2012). Participant's interbeat intervals were recorded during resting state and also assessed performance on the reading the mind in the eyes task (Baron-Cohen et al., 2001), an index of emotion recognition and theory of mind (TOM). As predicted, we reported that increased HRV was associated with better emotion recognition. Importantly, this was found even after accounting for a number of potential confounding variables (i.e., physical activity levels, age, sex, body mass index, smoking, depression, anxiety, and stress). An earlier study demonstrated that resting state HRV was associated with how quickly children with ASD can recognize emotions (Bal et al., 2010). Therefore, autonomic cardiac control plays an important role in directing resources for effective emotion perception.

A second prediction is that any change in physiological flexibility will correspond to a change in emotional expressivity or *projection* of emotions. The expression of emotional state is important as it signals what an individual may be thinking or feeling and also provides clues to future behavior. Both the perception and projection of emotion are essential for successful social communication as deficits in one or both of these domains will have detrimental impact on social communication. To test this prediction, researchers have coded facial expressions from video recordings and collected facial electromyography (EMG) measures in addition to ANS activity measures. Activity of the corrugator supercilii and zygomaticus major muscles, which can be observed with the naked eye—but more accurately measured by facial EMG—is a reliable index of negative (i.e., frowning) and positive (i.e., smiling) emotional expressions (Lang et al., 1993). Butler et al. (2006) examined HRV and facial expressions during a filmed dyadic interaction involving a distressing conversation over an upsetting war film that had been previously demonstrated to elicit strong negative emotions (Butler et al., 2003). Participants with higher resting HRV expressed greater negative (i.e., sad) emotion (indexed via coded video recordings of these interactions) during this conversation highlighting the importance of autonomic cardiac control in emotional response. The expressed negative emotions were described as typical, socially appropriate responses thus the authors interpreted these findings as a demonstration of HRV regulating a range of emotional reactions that are dependent on context, which in this case was a distressing topic. It was also predicted that individuals with poor vagal regulation (e.g., psychiatric populations) would demonstrate less socially appropriate responses. Another study on 5-month old infants reported that those with higher resting state HRV had greater facial expressivity (Stifter et al., 1989). The relationship between facial EMG and autonomic cardiac control has also been investigated in children. This research indicates that high HRV is related to greater facial expressivity in healthy children, but not children with disruptive behavior disorders (Marsh et al., 2008), consistent with other studies reporting poor empathic responding in this population (Herpertz et al., 2005). Similarly, Kettunen et al. (2000) have also explored HRV and facial expressions using EMG during Rorschach testing in adults. HRV at baseline and during the Rorschach task was associated with facial EMG measures. Consistent with the Polyvagal theory, the results of these experiments suggest that facial expressivity is related to the ANS. Thus, the CV system plays a vital role in how social cues are perceived and how social cues are projected to others (Gutkowska et al., 2000).

## The biology of oxytocin receptors in the cardiovascular system

OT is largely synthesized in the supraoptical and paraventricular (PVN) nuclei of the hypothalamus with direct OT projections to the dorsal brain stem, a crucial region for CV regulation (Buijs et al., 1978; Sofroniew and Schrell, 1981). OT receptors are distributed widely throughout the central and peripheral nervous system, with large concentrations in regions of the brain important for the regulation of complex social behaviors (Landgraf and Neumann, 2004). Animal research has established via radioimmunoassay that OT receptors are also located in the heart with the highest concentration located in the right atrium (Jankowski et al., 1998) in an amount comparable to the hypothalamus (Gutkowska et al., 2007). These OT receptors appear to confer their CV influence via their release of atrial natriuretic peptide (ATP) release (Gutkowska et al., 1997). Gutkowska et al. (1997) have proposed that the release of ATP via OT receptors is involved in the homeostatic regulation of blood volume. The detection of increased blood pressure signals OT release from the pituitary gland via baroreceptor input to the brain stem, which mediates the release of ATP in the right atrium. In turn, this activates the release of cyclic guanosine monophosphate (cGMP). The force of heart contraction is also reduced by the action of cGMP on cardiac myocytes and ATP on the right ventricle. In addition, OT may also influence autonomic cardiac control centrally via its input on the amygdala in particular (Domes et al., 2007b; Gamer et al., 2010; Labuschagne et al., 2010) In light of the widespread distribution of OT receptors within the CV system and in the neural structures that impact on this system, investigators have examined the impact of the peripherally administered of OT on a range of CV measures with more recent work focusing on HRV.

## The effect of oxytocin administration on cardiac function

Research suggests that OT administered intravenously decreases blood pressure in rats (Petty et al., 1985; Petersson et al., 1996), and administration over 5 days can reduce blood pressure for up to 2 months (Holst et al., 2002; Petersson and Uvnäs-Moberg, 2008). In humans, reduced blood pressure has been observed in women during labor following intravenous OT administration (Thomas et al., 2007; Sartain et al., 2008; Simpson and Knox, 2009). Central OT administration in animals has also been found to reduce stress-induced tachycardia (Morris et al., 1995). There have been mixed results for the impact of OT administration on HR. In animal models, OT administration has been found to increase (Mack et al., 2002), decrease (Mukaddam-Daher et al., 2001), or have no effect (Holst et al., 2002) on HR. Research in humans suggests that intranasal administration of OT has no impact on HR (Norman et al., 2011; Kemp et al., 2012a,b). A lack of change in HR after OT administration may be due to intranasal OT increasing activity of both branches of the ANS (Norman et al., 2011). OT is also involved in the neural modulation of breathing via a subpopulation of PVN cells that innervate motorneurons and neurons in the rostral ventrolateral medullary (Mack et al., 2002), a region involved in respiratory rhythm regulation (Ross et al., 1984).

Currently, it is unclear whether intranasal OT administration has a direct effect on peripheral ANS structures or whether its effects are mediated via central structures, which subsequently impact on the peripheral ANS. Intranasally administered OT reaches central structures via olfactory bulb pathways between the nasal mucosa and the brain (Bahadur and Pathak, 2012). The trigeminal nerve offers a potential pathway to the CNS for intranasally administered OT (Thorne et al., 2004; Guastella et al., 2012; Liu et al., 2012). This trigeminal pathway is interesting in the context of the autonomic cardiac control as the trigeminal nerve projects to trigeminal nuclei in the brainstem, with the principal sensory nucleus located lateral to the motor nucleus, a crucial region for cardiac autonomic regulation. As discussed above, there are direct pathways between brain stem regions controlling cardiac regulation and the site of central OT synthesis (i.e., the hypothalamus). Therefore, intranasally administered OT may mimic naturally synthesized OT by arriving at the same central site of action for autonomic cardiac control albeit via a different route.

## Oxytocin and heart rate variability

Recent research suggests that OT modulates HRV in both animals and humans. In an investigation of the impact of OT administration on HRV, Grippo et al. (2009) exposed prairie voles to 28 days of isolation or pairing with a sibling. The voles were administered either OT or placebo and exposed to behavioral stress. As expected, the isolated voles administered saline vehicle demonstrated decreased HRV after stress exposure. However, the isolated voles that were administered OT exhibited similar HRV to voles that were paired with siblings, indicating that OT may attenuate stress-induced reductions in HRV. Human trials have also explored the impact of OT administration on HRV. In a double-blind, between-subjects experiment Norman et al. (2011) examined parasympathetic and SNS function in response to OT administration. In addition to increasing PNS activity, OT administration also increased SNS activity, indexed by the pre-ejection period. These findings were also associated with self-report ratings of loneliness such that individuals that reported more loneliness were less likely to display increased PNS and SNS activity in response to OT administration. As OT is thought to promote pair bonding, the co-activation of both ANS branches is consistent with Paton et al. (2005) suggestion that reflexes associated with survival, such as the defense response (Koizumi and Kollai, 1981), tend to be associated with co-activation of the PNS and SNS whereas homeostatic reflexes are generally related to “classic” PNS/SNS reciprocal coupling. Berntson et al. (1991) have also suggested that coactivation of the PNS and SNS facilitates fine-grained control of the target organ's function whereas reciprocal PNS/SNS behavior is better suited for responses that require speed and magnitude, such as the baroreflex. Thus, dual activation of both branches of the ANS indicates that OT may be used to “fine-tune” social behavior (via autonomic cardiac control) rather than providing large and rapid responses that are better suited to functions related to homeostasis. In recent work, we acutely administered OT to humans (Kemp et al., 2012a,b) and focused on resting-state HRV. Participants were either given OT or placebo on their first visit, and returned 1 week later to receive the alternate intranasal spray. Forty-five minutes after administration a 10 min ECG was recorded from each of the participants. As predicted, OT administration increased HRV. Thus, human evidence suggests that OT administration can potentially increase autonomic regulation, increasing an individual's capacity for social engagement supporting our proposal that the effects of OT on social behavior and cognition may be, in part, mediated by the ANS.

## The social approach/avoidance hypothesis of oxytocin

There have been a number of hypotheses as to the central function of OT in humans including prosocial behavior (Tops, 2010) and social salience (Shamay-Tsoory, 2010). Recently, we (2011) proposed the social-approach/withdrawal hypothesis (Kemp and Guastella, 2011). This hypothesis suggests that OT increases social approach behaviors, which may be either positively (e.g., social cooperation and bonding) *or* negatively valenced (e.g., aggression and envy). Given the key role of cardiac function in social approach behavior (Kemp et al., 2012a,b), this theory is consistent with reports of increased HRV after OT administration (Norman et al., 2011; Kemp et al., 2012a,b) and reduced HRV in psychiatric disorders characterized by impaired social behavior (e.g., Kemp et al., 2010). In addition, decreases in HRV have been found to correspond to more avoidance-related behaviors such as defensiveness (Movius and Allen, 2005). Some have suggested that although the prosocial actions of OT are facilitated by approach behaviors that this is context dependent (Scheele et al., 2012) when one considers that OT is implicated in outgroup non-cooperation (De Dreu, 2012) and risk aversion (Declerck et al., 2010). However, both of these responses can be attributed to the “prickly” side of OT as defensive aggression may be considered an approach-related emotion. A number of studies on the impact of OT on trust also support this hypothesis. For example, Kosfeld et al. (2005) administered a single dose of OT or placebo to participants before they played a trust game and reported that those were given OT were more generous in giving money to others. Andari et al. (2010) also showed that OT enhanced social decision-making and trust in autism. In this experiment, participants with ASD were given either OT or placebo and played a simulated ball game with computer-simulated partners. Participants that were administered OT indicated greater feelings of trust and stronger cooperation with their most cooperative partner.

More recently, our social-approach/avoidance hypothesis has been further supported by work on pupil dilation (Leknes et al., 2012), which is also linked to ANS function. In this study, Leknes et al. reported that OT administration increases pupil dilation, which can influence approach-related behaviors via increased attractiveness of larger pupils (Wiseman and Watt, 2010) or facilitating greater interest toward rewarding stimuli (Laeng and Falkenberg, 2007; Bijleveld et al., 2009). In addition, research on OT and social stress is also consistent with the social-approach/avoidance hypothesis (Kubzansky et al., 2012). In this investigation, Kubzansky et al. administered either OT or placebo that was followed forty minutes later with the induction of social stress. Participants administered OT demonstrated a benign pattern of CV reactivity in comparison to the placebo group. They described that the group administered OT had a CV profile that was characteristic of the biological readiness to approach others in a stressful environment, supporting our social-approach/avoidance hypothesis.

## Oxytocin, autonomic cardiac control and social functioning in psychiatric disorders

Social functioning is impaired in a variety of psychiatric disorders and a number of studies have now shown that OT may help to resolve these impairments (Guastella et al., 2010; Averbeck et al., 2012; Pedersen et al., 2011). Research indicates that patients with autism—a disorder associated with significant social impairment—have low plasma OT levels (Modahl et al., 1998) and reduced HRV in comparison to healthy controls (Ming et al., 2005; Bal et al., 2010; Van Hecke et al., 2009). Similarly, research has shown that schizophrenia has lower levels of OT (Kéri et al., 2009), reduced HRV (Mujica-Parodi et al., 2005) and corresponding deficits in social cognition (Green et al., 2008).

OT administration improves social cognition in psychiatric patients, which may be facilitated, in part, by its impact on cardiac function. For example, we have demonstrated that single administration of OT in individuals with autism improves TOM (Guastella et al., 2010). OT has also been shown to improve emotion recognition with acute OT administration (Averbeck et al., 2012) and TOM with 14 days of chronic OT administration (Pedersen et al., 2011) in patients with schizophrenia. Intriguingly, the administration of OT increases gaze to the eye region of the face (Guastella et al., 2008), a candidate mechanism through which OT may enhance the recognition of facial expressions of emotion and social cognition more generally. In line with our initial hypotheses, more recent papers have shown than administration of OT increases gaze to the eye region in patients with autism (Berntson et al., 1997; Andari et al., 2010). The effects of OT on eye gaze may be a result of reduced amygdala activation (Kirsch et al., 2005), modulation of the visceral efferent pathways that regulate the striated muscles of the face and head, and increased vagal inhibition of the heart and bronchi (Porges, 2007, 2011). Our recent findings reporting an association between HRV and emotion recognition (Quintana et al., 2012) directly relates individual differences in HRV to emotion recognition, a core feature of social cognition. OT administration may also facilitate face-processing networks in the amygdala (Gamer et al., 2010) in addition to attenuating fear responses. Research shows that other disorders may also benefit from the administration of OT. Participants with social anxiety disorder administered OT as an adjunct treatment with exposure therapy were shown to improve their self-evaluations of a speech exposure task suggesting that OT augments the processing of positive social stimuli (Guastella et al., 2009). OT has also been shown to attenuate stress reactivity in borderline personality disorder (BPD), which may assist with emotion regulation (Simeon et al., 2011).

Although there have been some promising positive results from studies investigating intranasal OT and social behavior and cognition, findings suggest some important caveats. For example, although OT has been found to increase trust in healthy participants (Kosfeld et al., 2005), OT may hinder trust in individuals with BPD (Bartz et al., 2011a). Additionally, OT has been found to reduce trust if the other belongs to a social out-group that represents a threat (De Dreu et al., 2011; but see Chen et al., 2011). Some have argued that, rather than enhancing prosocial behavior, OT may increase the detection of social cues that are then filtered by existing psychopathology and contextual factors (Bartz et al., 2011a,b). We suggest that OT increases approach-related behaviors, which may be influenced by individual and contextual factors, via increased autonomic cardiac control. Thus, the interaction of OT with ANS should be taken into consideration when interpreting the effects of OT administration observed in psychiatric disorders.

## Conclusions and avenues for future research

While it has been established that OT assists with social cognition, research is yet to determine whether the effects of OT on autonomic cardiac control facilitate these increases in social cognition. We suggest that this may be the case. Vagal regulation and general systemic dysregulation clearly plays a role in the development and maintenance of many psychiatric disorders, and OT has demonstrated promise in the treatment of social dysfunction. While contradictory findings have been reported in regards to the beneficial effects of OT in psychiatric disorders (e.g. BPD), variation in reported findings may be due to interindividual differences in nasal anatomy that influences the deposition of intranasally administered OT (Djupesland, 2013; Guastella et al., 2012). In fact, little is understood on how intranasally administered OT impacts on central brain structures related to autonomic cardiac control or social cognition. Currently, the bioavailability of intranasally administered OT is unclear (Guastella et al., 2012). Only one study in humans has explored the central effects of an OT-like peptide (vasopressin) in response to intranasal administration (Born et al., 2002). The results of this study indicated that OT administration elevates OT in central spinal fluid rather than the periphery. Early work suggests that OT may be radiolabeled for the purpose of tracking the delivery of OT into the brain (Jelinski et al., 2002). To determine the bioavailability of intranasally administered OT, future research would benefit from the radiolabeling of OT in combination with positron emission tomography. Tracking radiolabeled OT administered intranasally would also help determine if OT reaches central structures that regulate cardiac autonomic control.

We conclude our review by highlighting that OT shows promise in the treatment of disorders characterized by poor social functioning. We suggest that the effects of OT may, in part, relate to the effects of OT on cardiac autonomic control as indexed by HRV. However, more research is needed on larger and more representative populations. Deficits of autonomic regulation will inhibit an individual's ability to appropriately approach or withdraw in social situations. Therefore, peripherally administered OT may benefit social interaction, in part, through its impact on cardiac autonomic control. Recent research showing that intranasal OT increases HRV in humans (Norman et al., 2011; Kemp et al., 2012a,b) provides support for this proposal. This research indicates that OT produces concomitant increases in SNS in addition to PNS activity suggesting that OT may promote a fine-grained control of cardiac autonomic control. If increases in social cognition via OT administration are underpinned by increased autonomic cardiac control then this offers a physiological marker of response to OT. We recommend that future work exploring the role of OT in social cognition should also consider measuring cardiac autonomic function.

### Conflict of interest statement

The authors declare that the research was conducted in the absence of any commercial or financial relationships that could be construed as a potential conflict of interest.
